# Calcium influx and sperm-evoked calcium responses during oocyte maturation and egg activation

**DOI:** 10.18632/oncotarget.19679

**Published:** 2017-07-29

**Authors:** Ya-Ru Xu, Wan-Xi Yang

**Affiliations:** ^1^ The Sperm Laboratory, College of Life Sciences, Zhejiang University, Hangzhou, China

**Keywords:** oocyte maturation, calcium influx, egg activation, calcium oscillation, calcium response

## Abstract

Under the guidance and regulation of hormone signaling, large majority of mammalian oocytes go through twice cell cycle arrest-resumption prior to the fertilized egg splits: oocyte maturation and egg activation. Cytosolic free calcium elevations and endoplasmic reticulum calcium store alternations are actively involved in triggering the complex machineries and events during oogenesis. Among these, calcium influx had been implicated in the replenishment of endoplasmic reticulum store during oocyte maturation and calcium oscillation during egg activation. This process also drove successful fertilization and early embryo development. Store-operated Ca^2+^ entry, acts as the principal force of calcium influx, is composed of STIM1 and Orai1 on the plasma membrane. Besides, transient receptor potential channels also participate in the process of calcium inwards. In this review, we summarize the recent researches on the spatial-temporal distribution of store-operated calcium entry components and transient receptor potential channels. Questions about how these channels play function for calcium influx and what impacts these channels have on oocytes are discussed. At the time of sperm-egg fusion, sperm-specific factor(s) diffuse and enable eggs to mount intracellular calcium oscillations. In this review, we also focus on the basic knowledge and the modes of action of the potential sperm factor phospholipase C zeta, as well as the downstream receptor, type 1 inositol 1,4,5-trisphosphate receptor. From the achievement in the previous several decades, it is easy to find that there are too many doubtful points in the field that need researchers take into consideration and take action in the future.

## INTRODUCTION

**Figure 1:**
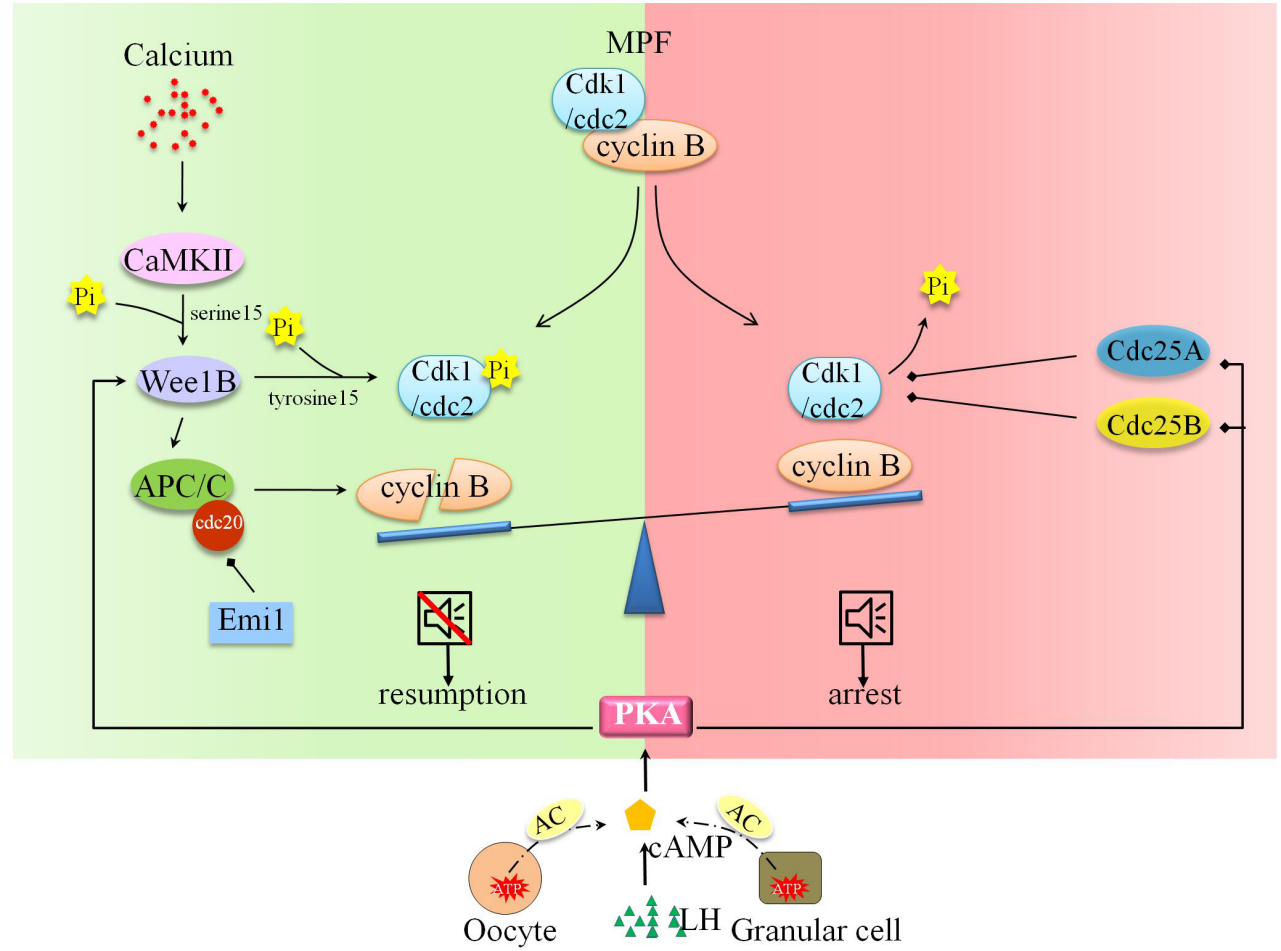
Molecular and signal mechanism underpinning oocyte arrest and cell resumption Oocyte arrest and resumption are balanced by two determinants: MPF and cAMP. The inactivation of MPF and regimentation of cAMP in arrested oocyte assist cell resumption. The phosphorylation status of Cdk1/cdc2 is activated by Ca^2+^-CaMKII-Wee1B pathway and inactivated by PKA-Cdc25A pathway. The degradation of cyclin B is promoted by Ca^2+^-CaMKII-Wee1B- APC/C pathway. What’s more, LH surge facilitates oocyte resumption *via* AC-cAMP-PKA pathway.

Oocytes in most mammal species undergo twice cell cycle arrests during cell meiosis. The first arrest happens at diplotene stage of meiosis I, termed germinal vesicle oocyte (GV oocyte). The second arrest appears at metaphase of meiosis II oocyte (MII oocyte). Under the stimulus of luteinizing hormone (LH), GV oocytes turn on the so-called “oocyte maturation” process, firstly detach from the arrest state, later undergo GV breakdown (GVBD) and endoplasmic reticulum (ER) reorganization. The oocytes accomplish the preparation for fertilization, finally terminate in MII stage. Subsequently, the ovulatory MII oocytes resume meiosis in response to sperm entry. The following events constitute the “egg activation” process: cortical granule exocytosis, polyspermy blockage *via* cortical reaction, meiosis II proceeding, genetic material replicating, second polar body (2PB) and pronuclei formation. Close attention had been paid to the molecular signaling basis of arrest exit downstream calcium (Ca^2+^) oscillation. The cytostatic factor, maturation-promoting factor (MPF), is composed of catalytic subunit cyclin-dependent kinase 1 (Cdk1)/cell division cycle protein 2 (cdc2) and regulatory subunit cyclin B. The inactivated MPF enforces Ca^2+^ command to call for cell cycle resumption. Two multiple interacting programs are responsible for the inactivation of MPF. On the one hand, the phosphorylation state of Cdk1/Cdc2 is coordinated by kinases and phosphatases. On the other hand, the synthesis and degradation of cyclin B are balanced. Reactivation and down-regulation of Wee1B exercised a great influence on Cdk1/Cdc2 kinase activity and pronucleus forming, and Wee1B inhibited Cdk1/Cdc2 activity by phosphorylating tyrosine 15 [1]. Wee1B activity could be activated by the phosphorylation effect of upstream type II Ca^2+^/calmodulin-dependent protein kinase (CaMKII) at Serine 15 [1]. At the other end of the scale are the downregulated phosphatases, like Cdc25A and Cdc25B, which could dephosphorylate Cdk1/Cdc2 and stabilize meiotic arrest [2, 3]. Activation of anaphase promoting complex/cyclosome (APC/C) and its co-factor Cdc20 (APC^cdc20^) could promote the proteolytic degradation of cyclin B and then inactivate MPF, finally release oocyte from MII arrest [4, 5]. What’s more, Wee1B could also mediate the degradation of cyclin B by activating APC/C [3]. The early mitotic inhibitor 1 (Emi1) could inhibit the activity of APC/C-APC^cdc20^ [6]. In addition to MPF, cyclic adenosine 3’,5’-monophosphate (cAMP) was also reported to maintain oocyte arrest [7, 8]. There are two sources of cAMP: the adjacent somatic follicular cells and the oocyte itself. In somatic follicular cells, cAMP could be catalyzed by adenylyl cyclase (AC) of ATP and transported into oocyte through gap junction [9]. In oocyte, G-protein-coupled receptors (GPCR) in the plasma membrane (PM) could activate AC3 to produce cAMP [10]. As the downstream of cAMP, protein kinase A (PKA) could balance the activities of the Wee1B kinase and Cdc25 phosphatase [11]. LH surge acts as a gap junction blocker to guarantee low concentration of cAMP in oocyte to assist cell resumption [12, 13]. The intricate and detailed signal interactions could be referred to many review papers [4, 14]. The signal pathways underlying oocyte arrest and resumption were depicted in Figure 1.

The inaugurator of MII oocyte resumption has been defined as a series of rapid, transient increase of intracellular calcium ( [Ca^2+^]_i_), known as “Ca^2+^ oscillation”, triggered by specific sperm factor. The successful cell cycle arrest and punctual exit are orchestrated by well-balanced [Ca^2+^]_i_ concentrations and complicated regulators/pathways, deeper issues needs to be solved in the future. The Ca^2+^ oscillation in mammalian eggs is featured by a train of long-lasting repetitive intracellular Ca^2+^ transients [15]. The frequency and duration of Ca^2+^ oscillations are different in various species. Take mouse eggs for example, the large transient increase lasts about 2 minutes, followed by repetitive transients lasting 0.5 minutes and occurring at 3 minutes intervals [16]. The Ca^2+^ oscillation in mouse eggs lasted until the pronuclear formation [17, 18]. Actually, the inordinate oscillatory pacemaker and Ca^2+^ oscillation during egg maturation and activation usually leads to egg-to-embryo transition disorders [16, 19].

The fine-adjusted [Ca^2+^]_i_ concentration is tightly and complicatedly regulated by various factors. It could be divided into the all-around administration of the Ca^2+^ entrance and exit. External Ca^2+^ ( [Ca^2+^]_e_) support Ca^2+^ influx and the authenticated channels that mediate Ca^2+^ influx during maturation and fertilization could be the type of plasma membrane channel(s), like store-operated Ca^2+^ entry (SOCE) and Cav3.2 [20, 21, 22]. Plasma membrane Ca^2+^-ATPase (PMCA) is a kind of Ca^2+^ pump-out channel that had been focused on. In presence of high concentrations of Gd^3+^, the initial [Ca^2+^]_i_ was broadened and the interval between spikes was widened, indicating the active participation of PMCA in [Ca^2+^]_i_ oscillations shaping [21]. ER plays a role as “Ca^2+^ reservoir” and is indispensable for Ca^2+^ oscillations. Replenishment and discharge of [Ca^2+^]_ER_ require sarco-endoplasmic reticulum Ca^2+^ ATPases (SERCA) and type 1 inositol 1,4,5-trisphosphate receptor (IP3R1) function during [Ca^2+^]_i_ oscillations. Addition of SERCA inhibitors (thapsigargin or cyclopiazonic acid (CPA)) could perturb Ca^2+^ oscillations by reducing basal [Ca^2+^]_ER_ levels and holding the recovery back [21, 23, 24].

As mentioned before, the fertilized Ca^2+^ fluctuation is stirred up by a specific sperm factor. The identity of such sperm factor was debatable and blurry over the past few decades, and many theories upgraded along with constantly deepened researches. Among these, phospholipase C zeta (PLCζ), was found to depend on the substrate phosphatidylinositol 4,5-biphosphate (PI(4,5)P2) to produce inositol 1,4,5-trisphosphate (IP3), and activate IP3R1 to release Ca^2+^ from ER store [25, 26, 27, 28]. Meanwhile, another controversial protein, postacrosomal sheath WW domain-binding protein (PAWP), made its first appearance in 2007 and disputes about its real function have cropped up repeatedly in recent years [29]. In this review, we summarized the findings in the past decades on the characteristics and the related signaling pathways underlying Ca^2+^ inward mobilization, such as ER-STIM1-Orai1 pathway [19, 30, 31], actin filament-TRPV3 pathway [32, 33], and TRPM7-SOCE pathway [34, 35, 36]. We also discussed the influence to oocyte maturation and activation. In addition, we described and discussed the sperm factors which operate Ca^2+^ release from ER during mammalian oocytes development and embryogenesis. The antagonists, inhibitors and labels used in the course of the study of Ca^2+^ are summed up in Table 1.

**Table 1:** Diverse pharmacological agents and/or materials used in the study of Ca^2+^ function in oocytes and eggs.

Name	application	Mechanism of action	demerits	references
2-APB	suppress intracellular Ca^2+^ release	inhibit IP3 receptor; inhibit SOCE	inequality in different cell types	[38, 115]
2-APB	TRPM7; activate TRPV3	unknown	-	[34, 35]
NS8593	inhibit TRPM7 channel	interaction between the pore-forming loop of TRPM7 and the imidazole ring of NS8593	Mg^2+^ dependence	[57, 116]
Naltriben	activate TRPM7 channel	most likely act via the TRP domain	-	[57, 117]
Sr^2+^	replace external Ca^2+^; induce parthenogenesis	potentiate the InsP3 receptors (InsP3R) without generating IP3; through the major route of TRVP3		[32, 65, 118]
BAPTA	calcium chelator		-	
fluo-3/8H	fluorescent calcium indicator		cause damage to oocytes and interfere with later embryo development	[37]
ionomycin	Ca^2+^ ionophore to release intracellular stores Ca^2+^	alter the plasma membrane permeability or act directly on intracellular organelles which release Ca^2+^	potentially cytotoxic or mutagenic	[119]
carvacrol	a TRPV3 agonist			[120]
heparin	competitive inhibitor of the inositol 1,4,5 trisphosphate receptor (InsP3R)		-	[20, 26]
Inp54p phosphatase	specifically dephosphorylate PI(4,5) P2		-	[91]
thapsigargin,	detect Ca^2+^ in ER stores	inhibit the ER Ca^2+^ ATPase (SERCA)	As the endogenous leak pathway is slow, thus as stores are being emptied, Ca^2+^ is buffered and extruded out of the cell	[38, 56]
A23187	Ca^2+^ ionophore to activate egg artificially		only elicit a single rise in Ca^2+^	[121]
thimerosal	induce [Ca^2+^]_i_ oscillations	sensitize IP3Rs without generating IP3	-	[122]
Cameleon D1ER	Ca^2+^ indicator	fluorescence resonance energy transfer (FRET)-based	-	[20, 37, 123]
Rhod-2	measure mitochondrial Ca^2+^/report cytoplasmic [Ca^2+^]_i_		-	[21, 69]
Gd3+	reduce Ca^2+^ influx and efflux	inhibit PMCA	-	[21, 124]
CPA	prevent [Ca^2+^]_ER_ increase	inhibit SERCA	-	[21]
mibefradil or/pimozide	inhibit CaV3.2	?	-	[22, 125]
TPEN	simulate store depletion	chelate store Ca^2+^		[19]

## Ca^2+^ INFLUX SUPPORTS ER STORES REFILLING, Ca^2+^ OSCILLATIONS AND DOWNSTREAM EVENTS OF EGG ACTIVATION

The existence of Ca^2+^ influx is vital for various physiological processes. When loading with IP3, neither mouse GV oocyte nor MII oocyte produced any Ca^2+^ release in Ca^2+^-free medium, but the Ca^2+^ oscillations are observed after Ca^2+^ addition [19]. The time cause of cation influx during fertilization-induced Ca^2+^ oscillations was evaluated by Mn^2+^ quenching method, and found that the internal flow augmented approximately 3-fold after each Ca^2+^ transient and gradually calmed down to the basal level until the next transient occurs in mouse eggs [37]. The functions of Ca^2+^ influx are versatile. It not only maintains Ca^2+^ oscillations by replenishing Ca^2+^ stores during oocyte maturation, but also provides an important spatially restricted Ca^2+^ signal required for complete egg activation at fertilization. For example, in intracytoplasmic sperm injection (ICSI) and/or *in vitro* fertilization (IVF) fertilized mouse eggs, Ca^2+^ influx across PM, instead of the intracellular Ca^2+^ buffering, is required for downstream events of egg activation [20, 37, 38]. They suspected that the Ca^2+^ influx through SOCE was a precondition for the successful IP3-mediated Ca^2+^ leak. The CaMKIIγ activation pathway was found as a downstream function cascade of Ca^2+^ influx in fertilized mouse oocyte as CA-CaMKIIγ cRNA-injected eggs formed a second polar body and pronucleus [20].

### Protein composition and molecular interactions of SOCE

SOCE is composed of two kinds of proteins: stromal interaction molecule 1 (STIM1) and Orail. STIM1, discovered in 1870, was characterized by malignancies/tumor-related function from human chromosome region 11p15.5 [39]. STIM proteins (STIM1 and STIM2), the single transmembrane-spanning proteins resident in the ER membrane, exist as dimers, and STIM1 migrates to the ER-PM junctions as “puncta” to tether Orai Ca^2+^ channels [40, 41]. The Ca^2+^ sensor role of STIM proteins in response to store depletion is dependent on its luminal single EF-hand Ca^2+^ binding domain [42]. The association of N-terminal domain leads to the unfolding and extension of the C-terminal domain, which is known as the STIM-Orai activating region (SOAR)/channel-activating domain (CAD)/Orai1-activating small fragment (OASF) [31, 43, 44, 45]. There are three Orai isoforms encoded in mammalian genomes. Orai3 resided and functioned in both the GV and MII oocyte membrane [19]. From the results of overexpression of Orai1, STIM1 or Orai3 in GV oocytes and MII oocyte, we could learn that, STIM1 associated with either Orai1 or Orai3 to mediate Ca^2+^ influx in the GV oocyte, but clustered with Orai1 only in the MII oocyte [19]. Orai1, a plasma membrane protein with four transmembrane domains, interacts with STIM1 by its cytoplasmic N- and C- termini to form the SOCE channel. The Ca^2+^ selective pore is composed of three layer construction by six Orai1 subunits: Ca^2+^ selectivity filter layer towards the extracellular end of the pore formed by glutamate residues that bind Ca^2+^ ions, the transmembrane helices (TM2-4) layer and the inner most transmembrane 1 (TM1) helices layer [40, 46, 47]. Recently, the gating mechanism of Orai1 by STIM1 was investigated, it occurred through a modest rotation of the pore helix, destroyed the V102-F99 hydrophobic band by shifting F99 residues away from the central pore axis, thereby increasing pore hydration and permitting ion conduction [41]. A more bewilderment, the signaling crosstalk underlies STIM1 recruitment of Orai1 and STIM1-Orai1 interaction remains enigmatic. In HEK293 cells, the phosphorylation of STIM1 at ERK1/2 target sites (i.e. in serine residues 575, 608 and 621) is necessary for the activation of SOCE [48]. In human pulmonary aortic endothelial (HPAE) cells, the phosphorylation at tyrosine residue 361 within the SOAR domain is a pivotal switch to link STIM1 puncta to gating of Orai1 channels [49]. From the perspective that two tyrosine residues lie within the SOAR domain of STIM1, the role of these potential phosphorylation sites in regulating oocytes and eggs SOCE remains to be disclosed.

### SOCE in oocyte maturation

In mammalian oocyte, whether SOCE exerts effects during maturation had been explored [30, 50, 51]. However, there is still a controversial dispute about the real occurrence of SOCE in maturing oocytes. Using the same thapsigargin and Ca^2+^ add-back method, Gómez-Fernández group reported that SOCE-mediated Ca^2+^ entry was silent in GV, GVBD and MI stage, increased sharply in MII oocyte [50]. Nevertheless, diametrically opposed to the above findings, Cheon group and Lee group insisted that Ca^2+^ influx decreased along with [Ca^2+^]_ER_ content increased, which showed the gradual inactivation of Ca^2+^ entry in mouse [19, 38]. In consideration of the inaccuracy of thapsigargin, Lee group verified their results through adopting a more direct approach to measure Ca^2+^ store by using the Ca^2+^ ionophore, ionomycin [19] (Table 1). The expression profile and subcellular localization of STIM1 and Orai1 disclosed the underlying molecular basis. For the former, quantitative real-time PCR and Western blot results revealed a low level of STIM1 at GV in the cortical fraction of the ER, followed by a sharp increase at the GVBD with homogeneously distribution throughout the periphery of the cell, but a steady expression of Orai1 with a scattered distribution in the plasma membrane, suggesting the decisive effect of STIM1 to the occurrence of SOCE [50, 52]. The low STIM1-Orai1 co-localization under store depletion conditions in immature oocytes suggested the close positive correlation between STIM1-Orai1 co-localization and SOCE activation during meiotic progression [50]. For the latter, the waning could be attributed to the progressively disabled SOCE, as the Ca^2+^ influx could be clearly enhanced in all stages of maturation by over-expressing of human-Stim1-YFP, especially in GV oocytes [38]. Interestingly, the cellular distribution of Stim1 and Orai1 changed during maturation, which coincided with the decline: STIM1 was patched throughout the GV oocyte, more diffuse in GVBD oocyte, more disperse as maturation progressed; Orai1 was highly enriched at the PM, weaker and internalized from PM to an intracellular early endosomal compartment [19, 38]. After ER store depletion, Orai1 translocated back to the cell membrane and coupled to an increase in STIM1 clusters [19]. What’s more, the ability of Stim1 to undergo “puncta” formation and migration to the cortex changed along with the decline in Ca^2+^ influx. The co-localization of hStim1 and hOrai1 diminished during this process [38]. Researchers in each group failed to provide a reasonable explanation for such discrepancy. There is speculation that the posttranslational (such as phosphorylation) and conformational modifications of STIM1 may exercise a great influence on SOCE activities. In HEK293 cells and human pulmonary aortic endothelial (HPAE) cells, the phosphorylation of STIM1 at several sites (i.e. in serine residues 575, 608, 621 and tyrosine residue 361) is necessary for the activation of SOCE [48, 49]. The auto-inhibitory domain was needed to expose by STIM1 to achieve full activation of SOCE [53]. What’s more, this brings into question the different physiological significance generated by the unusual distribution of SOCE components and this remains to be investigated.

Proper content of SOCE components has significant physiological effect to the follow-up activities and development. The expression pattern is shown up as higher in immature (GV-stage) oocytes and weak in MII mature oocytes [54]. Orai1 downregulation by injecting siRNA in porcine oocyte prior to maturation showed the abolished Ca^2+^, suggesting the key role of Orai1 in store-operated Ca^2+^entry [54]. In contrast, down-regulation of STIM1 or Orai1 by siRNA injection into porcine GV oocyte did not reduce the Ca^2+^ store content and Ca^2+^ oscillations in another research [55]. The difference may be explained by the existence of endogenous STIM1 and Orai1. However, overexpression of STIM1 and Orai1 in porcine oocyte disrupted the maintenance of the long-lasting Ca^2+^ signal and led to the fertilization failure [54, 55].

### SOCE in egg activation

The location of STIM1 changed during early fertilization in mouse oocytes. In resting MII oocytes, STIM1 co-localized with the ER marker calreticulin in the small and discrete areas or clusters, translocated to larger areas like PM concomitant with the intracellular Ca^2+^ stores emptying induced by TG/ionomycin/IVF [52]. The role for STIM1 and SOCE in the calcium signaling during early stages of mouse oocyte fertilization had been proven in these findings. Both protein expression (STIM1-CT and Orai1-NT) and SOCE inhibitors (SKF-96365 and 2-APB) failed to regulate the Ca^2+^ oscillations, which showed the rare contribution of SOCE in Ca^2+^ oscillation maintaining in fertilized mouse eggs [37]. In contrast, the expression of STIM1-CCb9 could enhance the rate of Mn^2+^ entry and augment the oscillation frequency, a better explanation was that STIM1 may assist the Ca^2+^ pump-mediated Ca^2+^uptake and the IP3R/Ca^2+^ channels-mediated pump-out [37]. Given that, SOCE can be chosen as a key maturity assessment indicator for mouse oocyte. However, Gd^3+^ and Synta66 (two SOCE inhibitors) could not prevent ICSI-induced Ca^2+^ entry, which indicated that the requisite Ca^2+^ entry may be supported by alternative Ca^2+^ influx channels [20].

In porcine eggs, SOCE was confirmed to be the only prerequisite to reload the intracellular stores and sustain the repetitive Ca^2+^ signal at fertilization. Different SOCE inhibitors (gadolinium, 3,5-bis (trifluoromethyl) pyrazole 2, tetrapandin-2) were employed into the porcine eggs, and the Ca^2+^ entry that was triggered by thapsigargin-induced store depletion was blocked, the fertilization Ca^2+^ signal ceased abruptly [55]. Injection of STIM1 siRNA to porcine oocyte prior to fertilization destroyed the characteristics of fertilization, as failing to generate repetitive Ca^2+^ signals and refill stores, perturbing embryo development [56]. And overexpressed h-STIM1 and h-Orai1 up-regulated the basal Ca^2+^ levels in mouse oocyte, nevertheless, over-expression of STIM1 had no effect to porcine oocyte which hinted the self-sufficiency of endogenous STIM1 function [19, 56].

Recently, the rule of SOCE in oocyte Ca^2+^ signaling was overthrown and the indispensable status of SOCE in Ca^2+^ entry and fertilization became controversial. Oocyte-specific conditional knockout (cKO) mice for STIM1 and STIM2 respectively, STIM1/2 double cKO mice, and Orai1-null mice were generated [57]. These three proteins-missing models showed no difference in ER Ca^2+^ stores or Ca^2+^ influx at GV stage, no difference in the pattern of Ca^2+^ oscillations after fertilization, and no difference in the normal fertility. All the above results proved the negligible effect of SOCE during oocyte fertilization [57]. This makes the definitive conclusions remain elusive and everything would get murkier and murkier. Whenever we have a new data point, with an unknown output value, we put it through the model and produce our expected output through constant exploration.

## THE CONTRIBUTION OF TRP CHANNELS TO Ca^2+^ PERMEATION

The Transient Receptor Potential (TRP) channels family proteins are composed of about 30 members. They can be divided into in six subfamilies: TRPC-”canonical”, TRPM-”melastatin”, TRPV-”vanilloid”, TRPA-”ankyrin”, TRPML-”mucolipin” and TRPP/PKD-”polycystin” [58, 59]. As cations conductors, TRP channels have weak sensitivity to voltage, and initiate a plethora of cellular changes in response to various stimuli such as osmolarity, pH, temperature, taste, pheromones and intracellular stimuli such as Ca^2+^ and phosphatidylinositol signal transduction pathways, and plant compounds [59]. Among them, TRPV3 and TRPM7 hold great significance for Ca^2+^ signaling in mammal oocytes and eggs.

### TRPV3

As a heat-sensitive protein, TRPV3 was firstly cloned and characterized in Keratinocytes [60]. It is composed by two coiled-coil domains in NH_2_ terminal, intermediate four predicted ankyrin domains and six putative transmembrane domains in C-terminal [60]. The functional expression of mouse PM-located TRPV3 had been investigated. It increased in accordance with the grade of maturity during oocyte maturation, showing concretely as measurable currents at GV, small at MI, and maximum at MII stage [32]. By means of voltage clamp and calcium imaging measurements of wild type and TRPV3-knock out mouse oocytes, TRPV3 was defined to mediate Ca^2+^ permeation and initiate egg activation [32]. Nevertheless, TRPV3-induced Ca^2+^ permeation meant nothing to fertilization-associated [Ca^2+^]_i_ oscillations in mouse [32]. Recently, 2-APB was confirmed to target TRPV3 selectively to increase [Ca^2+^]_i_ without affecting IP3R1 [33]. In the same study, the expression and/or function of TRPV3 were found to be regulated by actin microfilaments in mouse egg [33]. It could be attributed to the blockage of the recycling of TRPV3 channels to PM by the depolymerization of actin. In consideration of temperature sensibility, TRPV3 may protect the balance of Ca^2+^ regulation against extreme, hostile temperature milieu. There is still a need to figure out unknown endogenous direct activators and targets of TRPV3 in oocyte and egg in the future.

### TRPM7

Strontium is able to initiate oscillations in experimental operation. However, strontium was able to mount oscillations in TRPV3-lacking GV oocyte, indicating the fruitless contribution of TRPV3 and the possible commitment of TRPM7- and/or TRPM6-like channels to mouse GV oocyte [35]. Based on publicly available microarray data and electrophysiological evidence, TRPM7-like channels were picked up. TRPM7 has a large TRPM homology region (around 700 amino acids) in the N-terminal, followed by a TRP domain C-terminal to the transmembrane segments, and coiled-coil domain and serine/threonine kinase domain in the C-terminal [34]. TRPM7 homomers were identified to be functionally expressed in both GV oocytes and eggs, last until post-fertilization [35]. TRPM7 is involved in the ubiquitous Ca^2+^ influx pathway that regulates many physiological functions including oocyte maturation and fertilization. It was demonstrated to contribute significantly to spontaneous Ca^2+^ influx in GV oocytes and Ca^2+^ influx following fertilization in mouse eggs through pharmacological assessment of TRPM7 inhibitors (Mg^2+^ and NS8593) and activator (Naltriben) [35, 57]. TRPM7 knocking-out model satisfied the research requirement and more potential functions are discovered. TRPM7 was essential for normal embryonic development as trpm7 knock-out mice are embryonic lethal before E7.5 [61, 62]. The blockade of TRPM7 (trpm7 knocking-out) impaired normal pre-implantation development, delayed progression to the morula stage, and inhibited blastocyst formation [35]. The first priorities are to assess the essence, mechanism and magnitude behind these effects.

It’s worth noting that the connection between TRPM7 and SOCE should be considered when discussing TRPM7 effects. A recent research depicted an acute functional link between TRPM7 and SOCE for the first time. Researchers found that in chicken DT40 B lymphocytes, suppression of TRPM7 by pharmacological and molecular ways lessened SOCE while overexpression of TRPM7 rescued SOCE. TRPM7 possesses dual identity as both an ion channel which can depolarize cells and increase intracellular calcium and a kinase which can phosphorylate downstream proteins [34, 63, 64]. Interestingly, using kinase-deficient mutants, TRPM7 was proved to regulate SOCE through its kinase domain, might affect STIM indirectly by engaging another protein partner of STIM or Orai proteins [36]. This gave us a new facet of SOCE-TRPM7 interaction; the previous TRPM7-Ca^2+^ conditioning findings might result from the additional secondary, indirect support of SOCE *via* the kinase activity of TRPM7.

Along with the natural aging, physiological reaction of Ca^2+^ oscillations remains unchanged despite deterioration in the oocyte’s ability to replenish Ca^2+^ from the extracellular environment [65]. The cellular events underpinning the declining Ca^2+^ influx may attribute to the reduced/damaged mediated-channel, for instance, SOCE and TRPV3. Notably however, the unwounded Ca^2+^ oscillation could not guarantee intact fertilization response, the other downstream events may account for the senescence-mediated defects.

### Other potential channels mediate Ca^2+^ entry

In addition to SOCE and TRP family member proteins, there are still other potential channels that mediate intracellular Ca^2+^ regulation. The α1 subunit of the T-type channel Ca_V_3.2, encoded by Cacna1h, also supports the entry of Ca^2+^ meiotic maturation and developmental activation. The function of T-type channels had been investigated in oocytes previously [66, 67]. Ca^2+^/calmodulin-dependent protein kinase II (CaMKII) was found to target T-type channels to activate Ach-induced Ca^2+^ current [68]. Cacna1h-/- females have reduced litter sizes, Cacna1h-/- or Ca_V_3.2-pharmacological inhibited eggs have reduced total and ER Ca^2+^stores and turbulent Ca^2+^ oscillation patterns [22]. The exact mechanism of T-type channel regulation in oocytes and eggs requires further elucidation. Plasma membrane Ca^2+^-ATPase (PMCA) is a kind of Ca^2+^ pump-out channel that had been focused on. In presence of high concentrations of Gd^3+^, the initial [Ca^2+^]_i_ was broadened and the interval between spikes was widened, indicating the active participation of PMCA in [Ca^2+^]_i_ oscillations shaping [21]. ER plays a role as “Ca^2+^ reservoir” and is indispensable for Ca^2+^ oscillations. Replenishment and discharge of [Ca^2+^]_ER_ require sarco-endoplasmic reticulum Ca^2+^ ATPases (SERCA) and type 1 inositol 1,4,5-trisphosphate receptor (IP3R1) function during [Ca^2+^]_i_ oscillations. Addition of SERCA inhibitors (thapsigargin or cyclopiazonic acid (CPA)) could perturb Ca^2+^ oscillations by reducing basal [Ca^2+^]_ER_ levels and holding the recovery back [21, 23, 24].

As an energy production center, the contribution of mitochondria to Ca^2+^ waves propagation cannot be ignored. Suppressing mitochondrial function destroyed [Ca^2+^]_i_ vibration, blocked ER refilling of Ca^2+^ and thus enhanced [Ca^2+^]_i_ [21, 69]. ATP synthesis in mitochondria participated in [Ca^2+^]_ER_ refilling and normal oscillations maintaining, but there are other possibilities, including, just conceivably, it may uptake Ca^2+^ into the matrix [70].

Above all, the accomplishment of well-balanced Ca^2+^ oscillation during oocyte maturation and egg activation is achieved by the coordination of various types of channels. However, recent studies only rest on the enumeration of the phenomenon and the expression levels of different channels. All of this means it is imperative that more research should focus on the signal interactions and mechanisms involved in Ca^2+^ handling in the future.

## STRUCTURE AND FUNCTION OF PLCζ

The identity of the “sperm factor” was initially confirmed to be protein, then the Ca^2+^ releasing function was found to depend on IP3R to produce IP3, and therewith, Ca^2+^-sensitive PLC became the most suspicion [25, 26, 27, 71, 72]. The true face of “sperm factor” was unveiled by Saunders group in 2002; they analyzed the testis-derived expressed sequence tags (ESTs) and sought out the novel testis-specific smallest PLC isoform, PLCζ [28]. Complementary RNA (cRNA) and/or protein microinjection and depletion assay verified the physiological role for PLCζ in Ca^2+^ oscillations, egg activation and embryo development during mammalian fertilization [28, 73, 74]. What’s more, the direct evidence from the RNA interference transgenic mice further settled the status of PLCζ [75]. And ICSI failure patients showed reduced/absent expression of PLCζ1 in the sperm, such atypical PLCζ protein expression may be explained by protein degradation/discard during spermatogenesis, instead of genomic abnormalities, and the expression regulation during spermatogenesis still worth to study in the future [76].

### The structural and functional basis of PLCζ

As a smallest, constitutive mammal PLC isoform, PLCζ employs a distinct mechanism to potently induce Ca^2+^ release in eggs in contrast with other PLC family members. Grasping the mechanism basic of protein domains could pave the way for intense research. PLCζ possesses a tandem pair of EF hand-like domains in the N-terminal, extended X and Y domains in the center region and a C2 domain in the C-terminal, lacking of the pleckstrin homology (PH) domain in other PLC isoforms [28]. To examine the role of each region of PLCζ, a series of domain-deletion/mutation constructs were created. EF hand domains and C2 domain had a major impact for XY catalytic activity [77, 78, 79]. The XY linker (the intervening region separates the catalytic X and Y domains) in PLCζ stretches longer than PLCδ and owns the specific basic amino acid residues in the Y catalytic domain [28]. It was XY linker, instead of C2 domain, that possessed significant role in the targeting of PLCζ to PI(4,5)P2, which depending on the positive charge residues [78, 80]. The relatively conservative positive charge residues may attract the positive charged PI (4,5)P2. The sharp decrease of EC50 of PLCζ after deletion of both EF hands domains indicated Ca^2+^ sensor role of EF hands, and further, the fact that replacement of PLCζ-EF hands domain by PLCδ-EF hands domain weakened Ca^2+^ sensitivity stressed the more effective capacity of PLCζ-EF hands domain [78, 81]. Apart from the XY linker, EF-hand domain containing positively-charged residues is also responsible for the PLCζ-targeting to the membrane PI(4,5)P2 [82]. Evidence supporting its role came from the halfway diminished function from the absence of XY linker, and the damaged PI(4,5)P2 binding from sequential reduction of the net positive-charged residues in EF-hand [80, 82]. In addition, EF-hand domains could be the last arbiter for the species-specific differences in PLCζ (enzymatic properties and potency to elicit Ca^2+^ oscillations), proved by the exchanging human for mouse EF-hand assay [83]. Deletion/replacement of the C2 domain of PLCζ resulted in the inability to trigger Ca^2+^ oscillation in mouse eggs without PIP2 hydrolytic enzyme activity descend and Ca^2+^ sensitivity receding [77, 81]. Recently, a missense homozygous mutation had been found in PLCζ-C2 domain of two patients, Phe 489 converted from Ile. Such mutation resulted in the missing of PLCζ in sperm, turbulence of PLCζ after injection in mouse oocytes, disordering Ca^2+^ variation and early embryonic arrest [84]. The mode of C2 domain action may be realized by its PI(3)P/PI(5)P binding. On the one hand, such binding may help PLCζ keep silent before working, on the other hand, this may help PLCζ target the corresponding intracellular PIP2-containing vesicle. The post-translational modification is of great concern for PLCζ to attain the functional ability. Proteolytic cleavage at the linker region between X- and Y-domains belongs to that, the two fragments kept performance unless immunodepletion/affinity-depletion [79]. Figure 3 summed up the possible functions of each domain of PLCζ.

**Figure 2:**
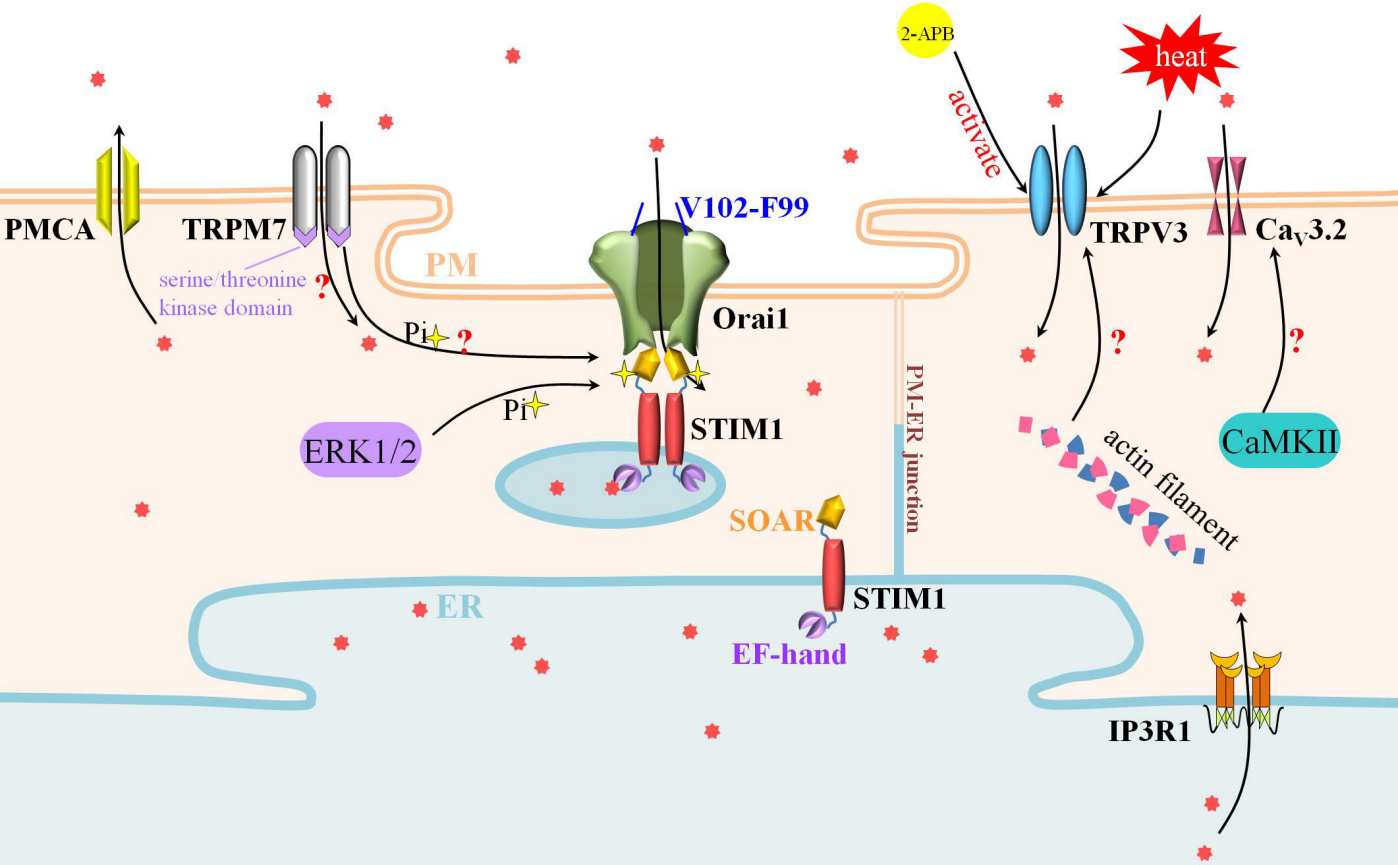
SOCE, TRPV3 and TRPM7 participate actively in Ca^2+^ influx during oocyte maturation and egg activation SOCE is composed of STIM1 and Orai1. STIM1 in the ER membrane tethered Orai1 in the PM to deliver the entrance of Ca^2+^ signal. Meanwhile, Ca^2+^ influx mediated by TRPV3 is crucial to oocyte maturation and activation. The polymerization of actin filament may promote the expression and PM-distribution of TRPV3. Besides the cation channel, TRPM7 could also phosphorylate SOCE by its serine/threonine kinase domain.

**Figure 3:**
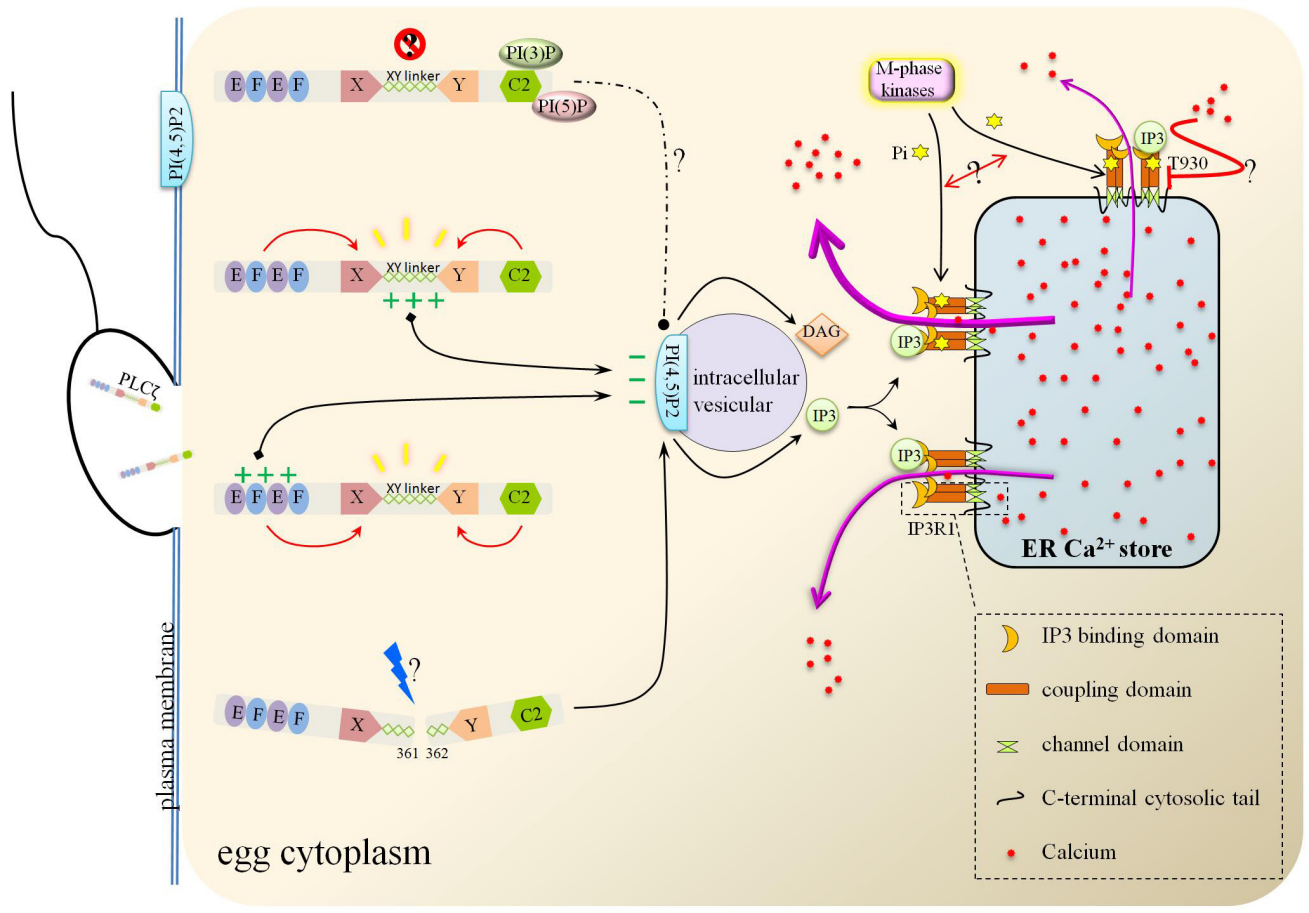
The action modes of PLCζ and Ca^2+^ release from ER stores Either XY linker or EF hand domain of PLCζ binds with the intracellular vesicular PI(4,5)P2 due to their positive charges. PI(4,5)P2 was then hydrolyzed to IP3 and DAG. As the ER membrane channel, IP3R1 mediated the Ca^2+^ flowing out from ER after binding with IP3. During this import process, M-phase kinases phosphorylated IP3R1 and promoted Ca^2+^ efflux.

The functional evaluation of PLCζ could be identified as an indication of the fertilizing ability of sperm and clinical diagnostic information. It could be assessed from two perspectives: the reduced/absent PLCζ protein abundance, the expressed mutated forms of PLCζ. The aberration of PLCζ expression like reduction or missing was detrimental for egg activation and the follow-up steps in embryo development [76, 85, 86, 87]. The impact of residue-mutated PLCζ on Ca^2+^ movement is also illustrated in the following examples. Sperms in two infertile brothers all had genetic missence homozygous mutations in PLCζ (Ile 489 Phe), which leading to the absence of PLCζ in sperm and mis-distribution after injecting into eggs/oocytes, without distorting the sperm morphology [84]. The histidine residue replacement of proline at position 398 and histidine residue replacement of leucine at position 233 of PLCζ were identified in a non-globozoospermic infertile male. Such mutations completely lack PIP2 (phosphatidylinositol 4,5-bisphosphate) hydrolysis ability *in vitro* and had lower protein instability when expressed in HEK293T cells [85, 88, 89, 90]. Above all, figuring out the structure-function relationships underlying PLCζ action is critical for understanding of the precise patterns of Ca^2+^ oscillations, oocyte activation and clinical applications.

Unlike other PLC isoforms in somatic cells, PLCζ targeted the internal PI(4,5)P2 residing distinct vesicular structures inside the egg cortex. Substantial evidences existed to support this, just like minimal loss of oolemma PI(4,5)P2, no affect of depletion of oolemma PI(4,5)P2 pool on PLCζ-mediated Ca^2+^ oscillations, immunolocalization of related proteins and the inhibition effect of targeted vesicular PI(4,5)P2depletion [91]. Such distinctive feature is in exact accordance with physiological composition of PLCζ, as lack of a PH domain which specific binding to PM-PI(4,5)P2, and the soluble property that supports PLCζ to diffuse throughout the cytoplasm. And even transforming the N-terminus of PLCζ by annexation of PH domain from PLCδ1 did not alter its *in vitro* biochemical properties [81]. What’s more, the distribution of PI(4,5)P2 also changed with the vesicular distribution of the Golgi and other membrane-trafficking systems. However, the exact domains mediate the binding with intracellular vesicles has remained a mystery.

PLCζ complementary RNA injection could arouse fertilization-like Ca^2+^ oscillations in mice, cows, pigs and humans, and the optimal concentration of PLCζ cRNA had been determined [74, 92, 93, 94]. These findings lay a solid foundation for future clinical reform and development to rescue human oocytes from failed activation. The active recombinant human PLCζ protein was obtained recently. Kashir group firstly purified recombinant human PLCζ protein and phenotypically rescued the failed activation in mouse oocytes, showing the great potential in clinical trials [88, 95]. Oocyte cytoplasmic movements was detected to change in near synchrony with the number and timing of Ca^2+^ transients after PLCζ injection in aged human oocytes that failed to fertilize after ICSI [96]. The temporal analysis of movements could be measured by particle image velocimetry (PIV) analysis [96]. It promises to be a non-invasive approach for assessing the Ca^2+^ oscillations pattern in oocytes.

### IP3R acts as PLCζ downstream pump-out channel on the ER membrane

Diacylglycerol (DAG) and IP3 are two products of PLCζ hydrolysis of PI(4,5)P2, IP3 then identifies and binds with IP3 receptors (IP3R), subsequently release the first Ca^2+^ wave from ER stores. IP3R1, tetrameric Ca^2+^ channels located on the membrane of ER, is responsible for the majority of [Ca^2+^]_i_ increases associated with fertilization [23, 24]. Such channel is composed of a channel pore formed by six transmembrane regions in C-terminal followed a small cytosolic tail, the coupling domain in intermediate region, and a ligand-binding domain in large cytosolic N-terminal region [97, 98]. IP3 is found to bind the coupling domain and could be converted into Ca^2+^ releasing from ER [99, 100]. Content and modification changes of IP3R1 are in accordance with the development of eggs. The level increased steadily with maturity, coinciding with maximal [Ca^2+^]_i_ oscillatory ability at the MII stage, lost over half of the receptors in pronucleus stage, corresponding to the sperm-initiated oscillations subsiding [101, 102, 103]. Persistent production and binding of IP3facilitated its degradation and its degradation-regulation contributed to shaping the pattern of sperm-initiated [Ca^2+^]_i_ oscillations [104]. In addition to IP3, Ca^2+^ can also act as a co-agonist [105]. On the other hand, phosphorylation of IP3R1 at the MPM-2 epitope by M-phase kinases (like p34cdc2 kinase, Cdk, MAPK and polo-like kinase-1 (Plk1)) enhances IP3R1-mediated Ca^2+^ release in mouse and porcine eggs [106, 107, 108, 109]. The phosphorylation is initiated around the GVBD stage, corresponding with the activation of these kinases, which are responsible for the initiation and progression of oocyte maturation. Strikingly, the specific phosphorylated residue T930 within the coupling domain during rat oocyte mitosis could lower the binding affinity of IP3 to IP3R and thus resulted in lessened IP3-dependent Ca^2+^ release [110]. The above paradoxical effects caused by phosphorylation of diverse residues implicates that the crosstalk and combinatorial fashion define the ultimate biological function of IP3R. The molecular and signaling pathways were showed in Figure 3.

### The controversial PAWP

PAWP, postacrosomal sheath WW domain-binding protein, locates in the post-acrosomal sheath region (PAS) of the perinuclear theca (PT) in elongating spermatids. It is composed of WW domain binding protein 2-similar homology in the N-terminal, PPXY consensus binding site for group-IWW domain-containing proteins, and numerous unique repeating motifs, YGXPPXG, in the C-terminal [29]. The performance of PAWP was tested by microinjection experiment and found that PAWP could promote MII oocyte meiotic resumption and pronuclear formation through its PY motif(s) [29].

While the effectiveness of PAWP to Ca^2+^ allocation is debatable, the battle of true identity of PAWP never ceased since its discovery. In 2015, two opposite research teams stated their points of view respectively in the same journal “Asian Journal of Andrology”, referring to “Is PAWP the “real” sperm factor?” and “Re: Is PAWP the ‘real’ sperm factor?” [111, 112]. Facing doubts and disproval from Aarabi group and other groups, Nomikos group made their utmost efforts to fight for their point of view rigorously and precisely. Even so, scientists carried out more intense studies through sufficiently rigorous programmes. Later then, depending on the PAWP-knockout mouse model, the veil of PAWP’s contribution to Ca^2+^ dynamics was further uncovered. Once again, the dispensable role of PAWP was supported by no indistinguishable changes in Ca^2+^ oscillations or in subsequent embryo development following gross depletion of PAWP [113]. The enigma that what PAWP does actually in other species on earth needs more exploration, maybe by means of new genome editing technologies, such as the CRISPR/Cas9 system.

## CONCLUSION AND PERSPECTIVES

Most mammalian oocytes undergo cell-cycle arrest and resumption for two times during meiosis, and changes in Ca^2+^ homeostasis play a significant role in these two processes. The regulation of intracellular Ca^2+^ homeostasis involves Ca^2+^ in-out of extracellular and intracellular stores. Amidst all of this, Ca^2+^ influx not only maintains Ca^2+^ oscillations by replenishing Ca^2+^ stores during oocyte maturation, but also provides an important spatially restricted Ca^2+^ signal required for complete egg activation at fertilization [20, 37, 38]. SOCE and TRP family proteins are dominating channels mediating Ca^2+^ influx. In this review, we analyzed the channel composition and molecular effectors, especially the basic knowledge and spatial-temporal changes of SOCE and TRP family proteins (Figure 2). Still, there are big divergences over the research data from different groups such as the distribution of STIM1 in the cause of oocyte maturation. SOCE is not the only channel to achieve Ca^2+^ afflux in mouse oocytes, but in porcine oocytes, there is still no substitute for SOCE. Interestingly, the cooperation of SOCE and TRPM7 provide the researchers the new mechanism of action [36]. To make matters more puzzling and complicated, with the advent of oocyte-specific conditional knockout mice, many researchers are confused to weigh the influences of SOCE to oocyte development. As mentioned above, proteins-missing models showed no difference with the control group in the Ca^2+^ regulation [57]. The hidden truth needs people to expose in the future.

In most mammal species, Ca^2+^ oscillations actually begin at the time when the sperm and egg fuses. One or a few materials from the sperm provoke the first release of Ca^2+^ from intracellular Ca^2+^ stores and propagate the Ca^2+^ waves. PLCζ is a candidate of sperm-specific factors. After releasing from sperm, PLCζ binds and hydrolyzes internal PI(4,5)P2 residing distinct vesicular structures into IP3 and DAG. As tetrameric Ca^2+^ channels located on the membrane of ER, IP3R1s take in IP3 and transform it into Ca^2+^ surge. Confronted with the intricate and multivariate factors in the action pathways, we went deep into and summarized the structural and modificatory regulation of PLCζ-induced Ca^2+^ oscillation (Figure 3). In this process there still has many mysteries waiting for us to explore. For example, the specific PLCζ antibodies used in different species and various laboratory protocols are still suspected along-standing puzzles. Multiple bands obtained from Western blotting could not definite the immunofluorescence or definite the position in sperms precisely, and in same species like human sperm, divergent results were received [76, 84, 85]. Therefore, the specificity and sensibility of the antibodies could not be neglected. Recently, Kashir group members exploited a novel, highly specific human PLCζ antibody and a uniform protocol (antigen unmasking/retrieval protocols), which might raise the accuracy in clinical and basic science study of PLCζ [114]. However, the feasibility and the universality still need more investigation, trials and tribulations. In the meantime, the true identity of PAWP is disputable since its discovery. Considering the limited overall evidence, more evidence of the links among PLCζ, PAWP and Ca^2+^ oscillations need to justified. More efforts should be redoubled to gain insight into the mechanism and molecular effectors that mediate the optimization of Ca^2+^ adjustment.

## Abbreviations

GV: germinal vesicle
MII: meiosis II
[Ca^2+^]_i_: cytosolic(intracellular) free calcium
[Ca^2+^]_ER_: endoplasmic reticulum Ca^2+^ store
SOCE: store-operated Ca(2+) entry
PM: plasma membrane
TRP: transient receptor potential
PLCζ: phospholipase C zeta
PAWP: postacrosomal sheath WW domain-binding protein
LH: luteinizing hormone
GVBD: GV breakdown
ER: endoplasmic reticulum
Ca^2+^: calcium
[Ca^2+^]_e_: external Ca^2+^
PMCA: PM Ca^2+^-ATPase
SERCA: sarco-endoplasmic reticulum Ca^2+^- ATPase
IP3R1: type 1 inositol 1,4,5-trisphosphate receptor
CPA: cyclopiazonic acid
PI(4,5)P2: phosphatidylinositol 4,5-biphosphate
IP3: inositol 1,4,5-trisphosphate
MPF: maturation-promoting factor
Cdk1: cyclin-dependent kinase 1
cdc2: cell division cycle protein 2
APC/C: anaphase promoting complex/cyclosome
CaMKII: type II Ca^2+^/calmodulin-dependent protein kinase
ICSI: intracytoplasmic sperm injection
IVF: *in vitro* fertilization
STIM1: stromal interaction molecule 1
